# Combining xQTL and genome‐wide association studies from ethnically diverse populations improves druggable gene discovery

**DOI:** 10.1002/alz70859_103217

**Published:** 2025-12-25

**Authors:** Noah J Lorincz‐Comi, Feixiong Cheng, Wenqiang Song, Xin Chen, Isabela Rivera Paz, Yuan Hou, Yadi Zhou, Jielin Xu, William Martin, Andrew A. Pieper, Jonathan L Haines, Chung Mina

**Affiliations:** ^1^ Cleveland Clinic, Cleveland, OH USA; ^2^ Cleveland Clinic Genome Center, Cleveland, OH USA; ^3^ Cleveland Clinic Foundation, Cleveland, OH USA; ^4^ Case Western Reserve University, Cleveland, OH USA

## Abstract

**Background:**

Progress toward identifying repurposed medicines to target Alzheimer’s disease (AD)‐associated genes has been hindered by a lack of viable candidate drug targets identified through genome‐wide association studies (GWAS). Gene‐based association tests provide a more statistically powerful alternative to standard inference based on detecting AD‐associated single nucleotide polymorphisms (SNPs), yet current approaches fail to leverage functional information from disease‐relevant tissues.

**Method:**

We developed a gene‐based association test (GenT) which can integrate summary multi‐omic data with large GWAS data to efficiently screen the genome for evidence of gene candidacy as an AD drug target. We leveraged brain cortex transcriptomic (eQTL) and proteomic (pQTL) data from ROSMAP, MetaBrain, and GTEx cohorts, as well as the largest AD GWAS to date, applying GenT to up to 18,273 genes. We experimentally evaluated one candidate in vitro using AD patient‐derived iPSC neurons.

**Result:**

We identified 151 druggable and potentially causal genes (e.g., RIPK2, NTRK1, RIOK1) associated with AD which were not within 1Mb of any known AD target from SNP‐based inference in GWAS. 103 were identified using eQTLs (e.g., ACE, BIN1, TNKS) and 74 by pQTLs (e.g., CR1, ABO, CLU), of which 26 were shared by both. Over 95% of candidate targets were replicated using SNP‐based causal inference from fine‐mapping with SuSiE. Experimental assays demonstrated that the NTRK1 protein inhibitor GW441756 significantly reduced tau hyper‐phosphorylation (including p‐tau181 and p‐tau217) in AD patient‐derived iPSC neurons, thus providing mechanistic support for our predictions.

**Conclusion:**

Our findings underscore the power and efficiency of gene‐based association testing, integrating multi‐omic information as a strategic and efficient tool for informed drug target discovery and validation based on human genetic and genomic data for AD and AD‐related dementia if broadly applied. These findings additionally highlight the limitations of standard inference based on detecting disease‐associated SNPs in GWAS, for which there is generally significantly less power than in gene‐based testing.

